# Expression Profile of Porcine TRIM26 and Its Inhibitory Effect on Interferon-β Production and Antiviral Response

**DOI:** 10.3390/genes11101226

**Published:** 2020-10-19

**Authors:** Hui Huang, Mona Sharma, Yanbing Zhang, Chenxi Li, Ke Liu, Jianchao Wei, Donghua Shao, Beibei Li, Zhiyong Ma, Ruibing Cao, Yafeng Qiu

**Affiliations:** 1 Shanghai Veterinary Research Institute, Chinese Academy of Agricultural Sciences, Shanghai 200241, China; huanghui0406@outlook.com (H.H.); monasharma1990@yahoo.com (M.S.); zhangyanbing129@outlook.com (Y.Z.); lichenxihsy@outlook.com (C.L.); liuke@shvri.ac.cn (K.L.); jianchaowei@shvri.ac.cn (J.W.); shaodonghua@shvri.ac.cn (D.S.); lbb@shvri.ac.cn (B.L.); zhiyongma@shvri.ac.cn (Z.M.); 2 College of Veterinary Medicine, Nanjing Agricultural University, Nanjing 210095, China

**Keywords:** TRIM26, antiviral response, IFN-β, poly (I:C), VSV, PRRSV

## Abstract

TRIM26, a member of the tripartite motif (TRIM) family has been shown to be involved in modulation of innate antiviral response. However, the functional characteristics of porcine TRIM26 (porTRIM26) are unclear. In this study, we used a synthesized antigen peptide to generate a polyclonal antibody against porTRIM26 with which to study the expression and function of porTRIM26. We demonstrated that polyinosinic:polycytidylic acid (poly (I:C)) stimulation and viral infection (vesicular stomatitis (VSV) or porcine reproductive and respiratory syndrome virus (PRRSV)) induce expression of porTRIM26, whereas knock-down expression of porTRIM26 promotes interferon (IFN)-β production after poly (I:C) stimulation and virus infection (VSV or PRRSV). The importance of the porTRIM26-mediated modulation of the antiviral response was also shown in VSV- or PRRSV-infected cells. In summary, these findings show that porTRIM26 has an inhibitory role in IFN-β expression and the antiviral response.

## 1. Introduction

Tripartite motif (TRIM) proteins, a large family of ubiquitin E3 ligase, include more than 80 and 60 members in human and mouse, respectively [1]. Moreover, more than 50 members of porcine *TRIM* genes have been annotated in the GenBank database, although most of them were predicted with computational analyses [2,3]. Recent studies have shown that many members of the TRIM family are expressed in response to interferons (IFNs) and are involved in the processes of innate immune response, especially during viral infections [4,5,6]. These reports strongly suggest that understanding the molecular functions of porcine TRIM proteins could offer insights into the regulation of innate immune response in swine species.

TRIM26 is a member of the TRIM family, and has a structure similar to that of many other members of the family. They are all structurally characterized by a RING finger domain (E3 ligase with ubiquitin ligase activity) with two B-box domains, followed by a coiled-coil (CC) region and a C-terminal protein binding domain [7,8,9]. In human, *TRIM26* gene is located in the MHC class I region [10,11]. Likewise, the porcine MHC (swine leukocyte antigen (SLA)) region contains *TRIM26* gene according to a sequencing analysis. However, although porcine *TRIM26* (*porTRIM26*) gene has been identified [2,12,13], the biological functions of the protein, especially in the immune response, remain to be determined.

Previous studies in human and mouse cell lines have shown that TRIM26 plays a controversial role in the regulation of IFN-β production and innate antiviral response, which may be attributable to the different experimental systems used [14,15]. However, how porTRIM26 affects the regulation of IFN-β expression and the innate antiviral response is unknown. In this study, for the first time, we determined the expression profile of porTRIM26 in different pig tissues and clarified its effect on the modulation of IFN-β expression and innate antiviral response.

## 2. Materials and Methods 

### 2.1. Tissue Sample Collection

Piglets (Shanghai great white pig strain; ~30 days old) were purchased from the Shanghai Academy of Agricultural Sciences (Shanghai, China), euthanized, and dissected to obtain various tissue samples (liver, spleen, lung, kidney, submandibular lymph node, hilar lymph node, mesenteric lymph node, inguinal lymph node, and thymus). The tissue samples were immediately snap-frozen in liquid nitrogen and stored at −80 °C. All animal experiments were approved by the Institutional Animal Care and Use Committee of Shanghai Veterinary Research Institute, Chinese Academy of Agricultural Sciences, Shanghai, China (IACUC No: Shvri-po-2016060501) and were performed in compliance with the Guidelines on the Humane Treatment of Laboratory Animals (Ministry of Science and Technology of the People’s Republic of China, Policy No. 2006398).

### 2.2. Cells, Viruses and Infections

Porcine alveolar macrophages (PAM) were generated as shown in our previous study [16]. HEK 293T cells, porcine iliac artery endothelial cells (PIEC), ST cells, PK-15 cells, BHK-21 cells, and Marc-145 cells were maintained in Dulbecco modified Eagle medium (Thermo Fisher Scientific, Shanghai, China) supplemented with 10% fetal bovine serum (Thermo Fisher Scientific, Shanghai, China) at 37 °C in a 5% CO_2_ atmosphere. Vesicular stomatitis virus (VSV) and highly pathogenic porcine reproductive and respiratory syndrome virus (HP-PRRSV) were maintained in our lab and were propagated on BHK-21 cells and Marc-145 cells, respectively. The viruses were titrated with the median tissue culture infective dose (TCID_50_) methods, as described in a previous study [17]. According to the progress of virus infection and expression of porTRIM26, we chose 24 h after infection for collecting the viral infected samples: PIEC cells were infected with VSV at a multiplicity of infection (MOI) of 1 for 24 h, at which point peak titer was reached with the induction of porTRIM26; PAM were infected with PRRSV at a multiplicity of infection (MOI) of 1 for 24 h, at which point peak titer was reached with the induction of porTRIM26. 

### 2.3. Cloning and Sequence Analysis of porTRIM26

The primers for cloning *porTRIM26* gene (shown in Table S1) were designed based on the *porTRIM26* gene sequence (GenBank accession number, NM_001123209.1). The amplified sequence was confirmed with DNA sequencing and inserted into the p3×Flag-CMV-14 vector (Sigma, St. Louis, MO, USA) to generate a recombinant plasmid expressing FLAG-tagged porTRIM26 (pFlag-porTRIM26). An amino acid sequence alignment of the deduced protein sequence by this construct and the TRIM26 proteins of another three species, *Homo sapiens* (NP_001229712.1), *Mus musculus* (NP_001020770.2), and *Rattus norvegicus* (XP_008770914.1), was conducted using the software Lasergene version 7.1 (Madison, WI, USA). A phylogenetic tree based on the sequences of different species was constructed by the neighbor-joining method using the MEGA software (version 6.06).

### 2.4. Generation of Polyclonal Antibody Against porTRIM26

A polyclonal antibody directed against porTRIM26 was generated as described in a previous study [18]. Briefly, a peptide of 20 amino acids corresponding to residues 364–383 of the porTRIM26 sequence was synthesized chemically and conjugated with keyhole limpet hemocyanin (KLH) as the carrier protein. Rabbits were immunized five times with the peptide-KLH conjugate combined with complete or incomplete Freund’s adjuvants. HEK 293T cells were then transfected with the porTRIM26-expressing recombinant plasmid described in Section 2.3 and a Western blotting analysis was performed to confirm the specificity of the polyclonal antibody. All of the animal experiments were approved by IACUC in Shanghai Veterinary Research Institute, CAAS (No: Shvri-po-201606 0501) and followed the guidelines described in Section 2.1. 

### 2.5. Plasmid Transfection, Small Interfering RNA (siRNA), and Polyinosinic:polycytidylic Acid (poly (I:C)) Stimulation

Cells were grown to 70–80% confluence and transfected with the plasmids expressing FLAG-porTRIM26 or the empty vector (p3×Flag-CMV-14 vector, as a negative control) with Lipofectamine 2000 (Thermo Fisher Scientific, Shanghai, China), according to the manufacturer’s instructions. After 24 h of transfection, PIEC cells were transfected with poly (I:C) at a final concentration of 3 µg/mL (InvivoGen, Toulouse, France) and stimulated for 6 h, at which *porTRIM26* and *IFN-β* was obviously induced according to a multiple time-point sample analysis by qPCR. At 6 h after stimulation, the samples were collected for further analysis. 

To investigate the role of porTRIM26, one porTRIM26 specific siRNA out of 4 (the targeted sequence: GCCTGTACCAGAGCTCTTA) was selected and transfected into PIEC cells or PAM by Lipofectamine RNAiMAX (Thermo Fisher Scientific, Shanghai, China) following the manufacture’s instructions. Likewise, a scrambled siRNA (sequence: TTCTCCGAACGTGTCACGT) was transfected as the negative control (NC). After 72 h of transfection, cells were treated with poly (I:C): the PIEC cells were treated as described above, and PAM were stimulated with poly (I:C) at a final concentration of 3 µg/mL without transfection. At 6 h after stimulation, the samples were collected for further analysis. 

### 2.6. Western Blottting Analysis

The protein samples were prepared as previously described [19]. In brief, the membranes transferred with the protein samples were blocked with 5% skim milk for 1 h at room temperature. The membrane was incubated overnight at 4 °C with the individual primary antibodies (anti-Flag (1:1000, M2, Sigma), anti-porTrim26 (1:1000, generated in this study), anti-VSV G (1:1000, Abcam), anti-PRRSV N (1:1000, generated by a synthetic peptide of N), and anti-actin (1:10,000, Sigma)). The membrane was then incubated for 1 h at room temperature with the secondary antibodies: horseradish-peroxidase-conjugated goat anti-mouse IgG (1:5000, Abcam) or goat anti-rabbit IgG (1:10,000, Abcam).

### 2.7. Enzyme-linked Immunosorbent Assay (ELISA)

The concentrations of porcine IFN-β in supernatants from PIEC cell culture or PAM culture were measured by ELISA kit (Lengton, Shanghai, China).

### 2.8. Reverse transcription (RT)-quantitative PCR (qPCR) Analysis

Total RNA was extracted with RNAiso Reagent (Takara, Dalian, China) and the cDNA was prepared with PrimeScript RT Reagent Kit (Takara, Dalian, China). Gene expression was analyzed by RT-qPCR using SYBR Green qPCR Master Mix (Takara, Dalian, China). The specific primers are shown in Table S1. The expression of the glyceraldehyde 3-phosphate dehydrogenase gene (*GAPDH*) was used as the reference. Expression was calculated relative to that of *GAPDH (2^−ΔCt^)*.

### 2.9. Statistical Analysis

All data were analyzed with GraphPad Prism software (Graphpad Software, Inc, La Jolla, CA, USA). An unpaired Student’s t-test was used to determine significant differences. Values were considered statistically significant when *p* < 0.05. Data were given as mean ± SEM as indicated; ‘n’ refers to the sample size.

## 3. Results

### 3.1. Sequence Analysis and Generation of Polyclonal Antibody Against porTRIM26

**Figure 1:**
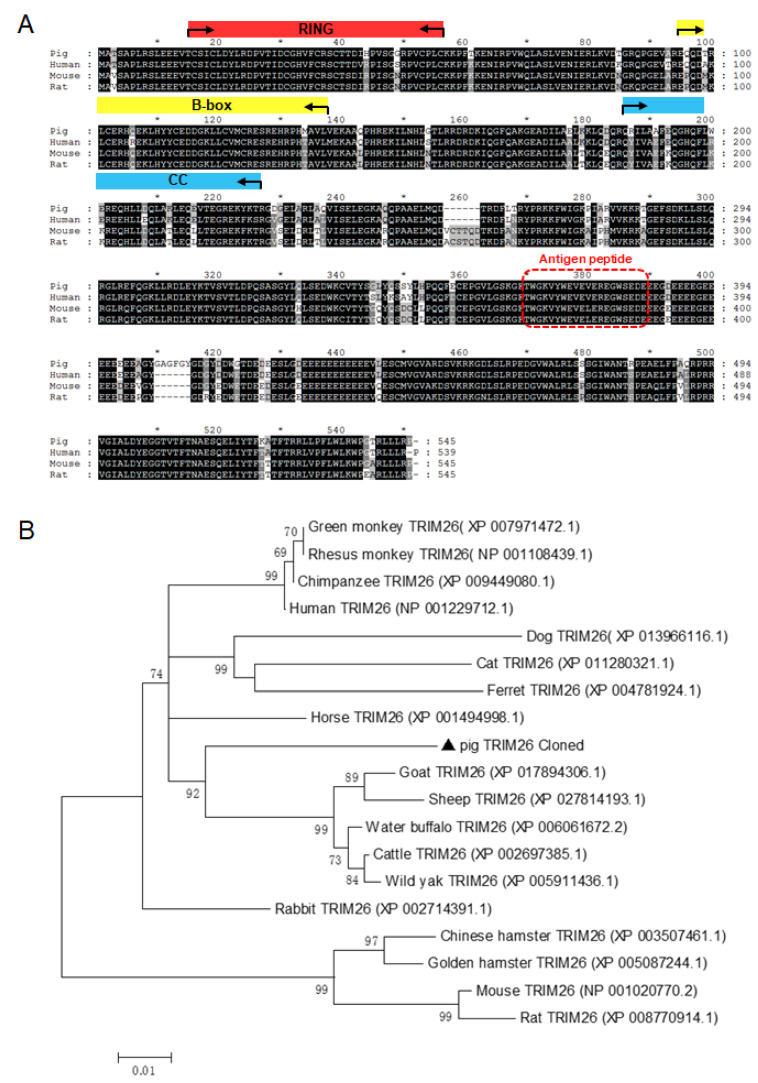
Multiple alignment and phylogenetic analysis of TRIM26. (**A**) Polypeptide of porcine TRIM26 was aligned with that of human, mouse, and rat. The RING finger domain (RING), the B-box domains (B-box), and the coiled-coil region (CC) are labeled in reference to the human TRIM26. The antigenic peptide is shown in the red box. (**B**) The phylogenetic tree of TRIM26, constructed by neighbor-joining method using MEGA 6.06.

Based on the porcine *TRIM26* gene sequence (GenBank accession number NM_001123209.1), the porcine *TRIM26* gene was amplified with RT-PCR from the total RNA extracted from pig lungs (Shanghai great white pig strain). A sequence analysis with BLAST showed that the cloned gene sequence from Shanghai great white pig strain was identical to the porcine *TRIM26* gene sequence in GenBank. The full-length cDNA of porcine *TRIM26* contained 1638 bp and encoded a protein of 546 amino acid residues including one Ring domain, two B-box domains, one coiled-coil domain, and one C-terminal domain, according to the protein BLAST information. A BLASTp analysis in the National Center for Biotechnology Information (NCBI) nonredundant database detected more than 200 TRIM26 orthologues (>50% identity) from more than 100 species. Notably, among the mammalian sequences, human and mouse TRIM26 shared 91% identity and 84% identity with porTRIM26, respectively, which is consistent with the data generated with the Clustal W method (Figure 1A). A phylogenetic analysis was performed to investigate the difference in the identities with TRIM26 of the other species. The *porTRIM26* gene clustered on a separate branch of the dendrogram within the sequences of Mammalia and was phylogenetically closest to the human sequence than to the mouse sequence (Figure 1B).

**Figure 2:**
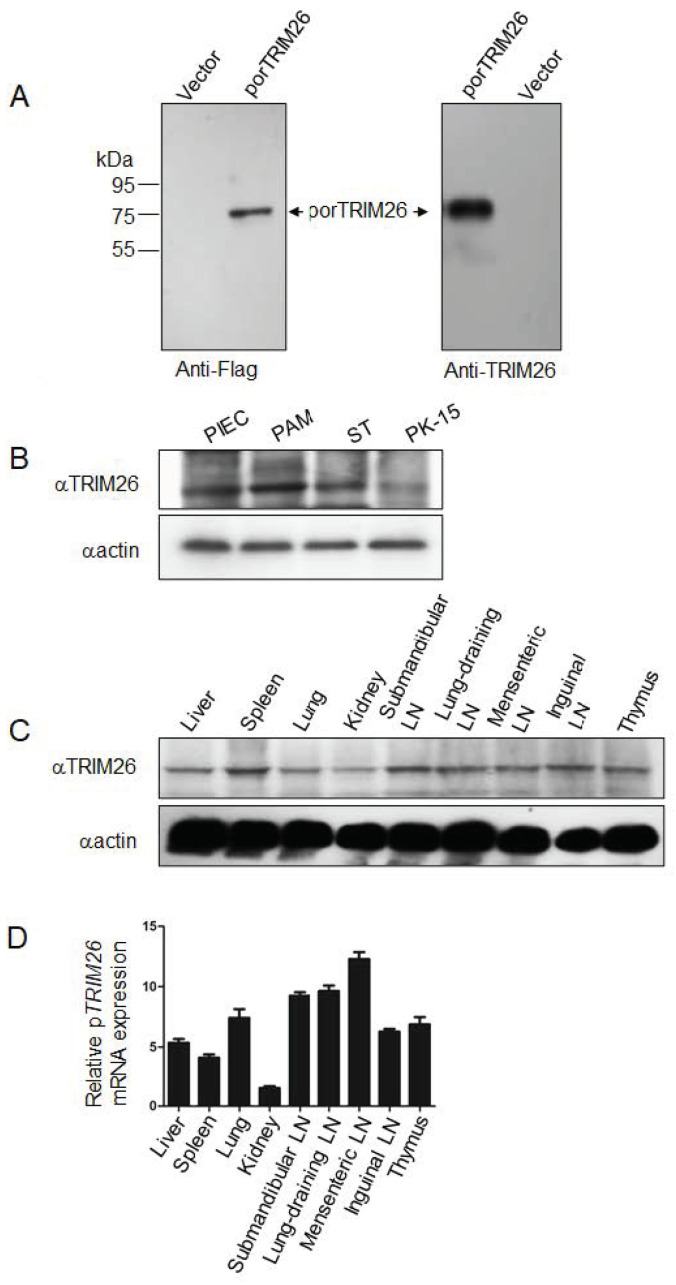
(**A**) Specificity of polyclonal antibody against porcine TRIM26 was analyzed with Western blotting. (**B**) Expression profiles of TRIM26 in different porcine cell lines, analyzed with Western blotting with anti-TRIM26 antibody. (**C**) Expression profiles of TRIM26 in different pig tissues (*n* = 3), analyzed with Western blotting and an anti-TRIM26 polyclonal antibody. Approximately 100 mg of each tissue was homogenized with lysis buffer as per the Materials and Method section for protein sample preparation. (**D**) Expression profiles of *TRIM26* in different pig tissues (*n* = 3) analyzed by reverse transcription-quantitative PCR.

To study porTRIM26 expression in different tissues and cells of pigs, we developed an anti-porTRIM26 antibody after synthesizing an antigen peptide (Figure 1A). We tested the specificity of the antibody with a Western blotting analysis. Our result indicated that the anti-porTRIM26 antibody specifically detected the expression of porTRIM26 in HEK 293T cells (Figure 2A), and that the signal was equal to that detected with an anti-Flag antibody.

### 3.2. Expression Profiles of porTRIM26 in Different Cell Lines and Tissues

The polyclonal antibody was used to determine the expression profiles of porTRIM26 in different porcine cell lines and tissues in a Western blotting analysis. First, the expression of porTRIM26 in different porcine cell lines was determined by Western blotting. The PK15 cell line had a lower level of porTRIM26 in comparison to that in other cell lines (Figure 2B). The expression of porTRIM26 in different tissues was also determined with Western blotting. In almost all examined tissues porTRIM26 was differentially expressed, including in liver, spleen, lungs, kidneys, lymph nodes, and thymus (Figure 2C). The lowest expression of *porTRIM26* was observed in the kidneys in RNA level (Figure 2D), consistent with its expression in protein level (Figure 2C).

### 3.3. porTRIM26 Negatively Regulates Expression of IFN-β

**Figure 3:**
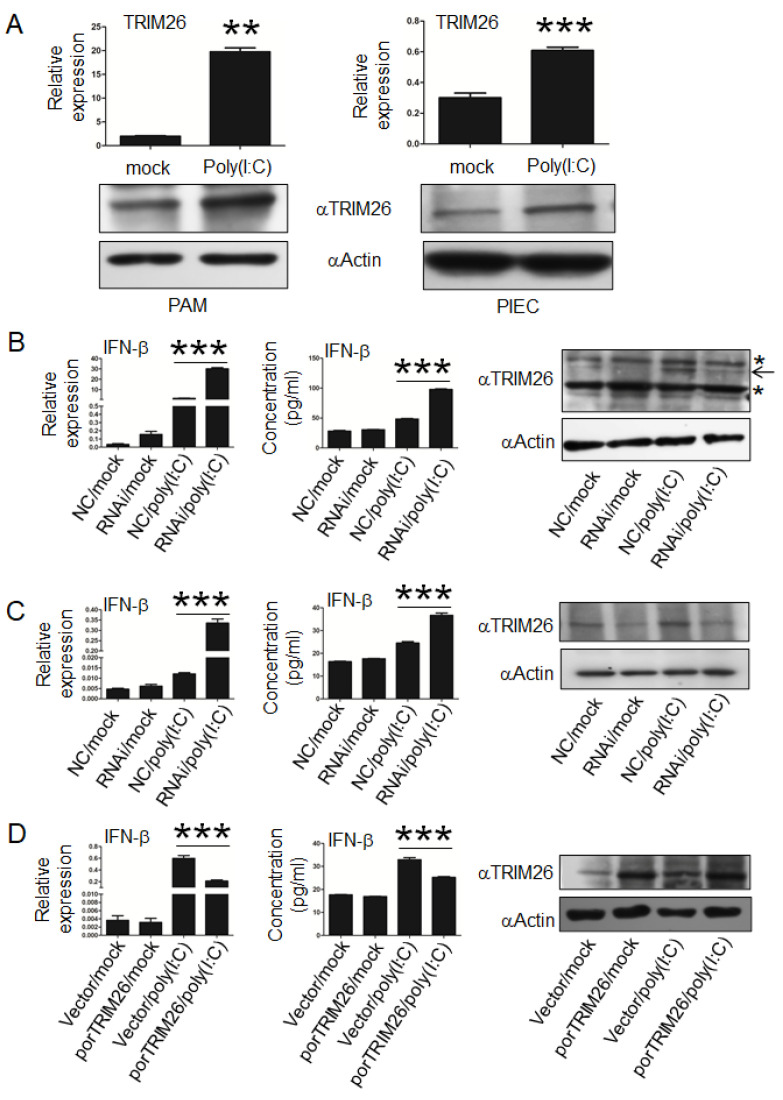
Effect of porTRIM26 on poly (I:C)-induced interferon (IFN)-β. (**A**) Expression of TRIM26 in porcine alveolar macrophages (PAM) or porcine iliac artery endothelial cells (PIEC) with or without poly(I:C) treatment was measured with qPCR and Western blotting. Poly (I:C) treatment induced the expression of TRIM26 in both PAM and PIEC cells. (**B**) PAM were transfected with porTRIM26 siRNA or negative control (NC) siRNA for 72 h and then stimulated with poly (I:C) for 6 h. Supernatant and cells were harvested and analyzed by enzyme-linked immunosorbent assay (ELISA) and qPCR, respectively. (**C**) PIEC cells were transfected with porTRIM26 siRNA or NC siRNA for 72 h, and then stimulated with poly (I:C) for 6 h. Supernatant and cells were harvested and analyzed by ELISA and qPCR, respectively. (**D**) PIEC cells were transfected with plasmid encoding porTRIM26 or the empty vector. After 24 h, the cells were treated with poly (I·C) or vehicle and incubated for another 6 h. Level of *IFN-β* mRNA was determined with RT-qPCR. Data present are mean ± SEM pooled from one independent experiment; *n* ≥ 3 for each of the analyzed parameters. *, *p* < 0.05; **, *p* < 0.01; ***, *p* < 0.001 in comparison between mock and poly (I:C) group (**A**); between NC and RNAi with poly (I:C) stimulation (**B**,**C**); and between the empty vector and pFlag-porTRIM26 with poly (I:C) stimulation (**D**).

The role of porTRIM26 in the regulation of IFN-β expression is controversial, which may be attributable to the different experimental systems, such as different cell types used in different studies. Whether porTRIM26 affects IFN-β production, either positively or negatively, remains unclear. We first investigated its effect on poly (I:C)-induced IFN-β expression using two kinds of porcine cells, PIEC cells and PAM. Notable, poly (I:C) stimulation induced the expression of porTRIM26 in both porcine cell lines (Figure 3A), consistent with the previous report in human and mouse cell lines [15]. To investigate the effect of porTRIM26 on IFN-β expression, a porTRIM26-specific siRNA was designed and used to transfect PIEC cells and PAM. A Western blotting analysis showed that the transfection of the siRNA reduced the expression of porTRIM26 (Figure 3B,C). The transfection of porTRIM26 siRNA promoted poly (I:C)-induced IFN-β expression in both PAM and PIEC cells (Figure 3B,C). In contrast, the overexpression of porTRIM26 in PIEC attenuated the poly (I:C)-induced IFN-β expression (Figure 3D). Collectively, these results reveal that porTRIM26 modulates IFN-β expression downstream of poly (I:C)-stimulated innate signaling.

### 3.4. porTRIM26 Negatively Regulates Antiviral Response to VSV Infection

**Figure 4:**
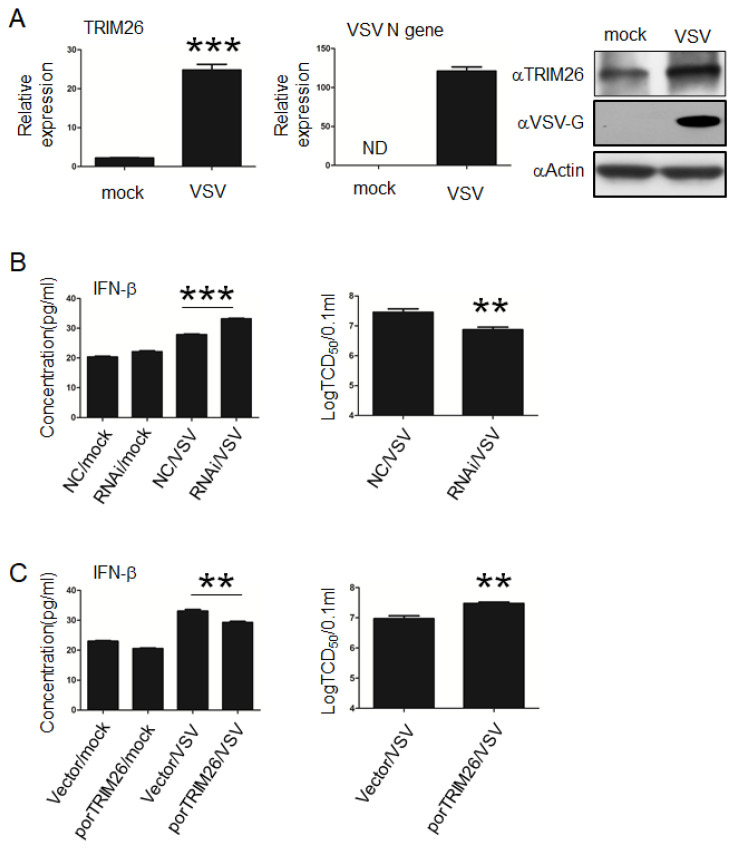
Effect of porTRIM26 on IFN-β expression and vesicular stomatitis virus (VSV) infection in PIEC cells. (**A**) PIEC cells were infected with VSV at a multiplicity of infection (MOI) of 1 for 24 h. The porTRIM26 mRNA and protein level was measured by RT-qPCR and Western blotting, respectively. (**B**) PIEC cells were transfected with porTRIM26 siRNA or NC siRNA. After 72 h, the cells were mock infected or infected with VSV at a MOI of 1. Supernatants were collected at 24 h post infection (hpi) for tissue culture infective dose (TCID_50_) assay. (**C**) PIEC cells were transfected with plasmid encoding porTRIM26 or empty vector for 24 h and then infected with VSV at an MOI of 1. Supernatants were collected at 24 hpi for either a TCID_50_ assay or ELISA. Data are mean ± SEM pooled from one independent experiment; *n* ≥ 3 for each of the analyzed parameters. ND, no detected. **, *p* < 0.01; ***, *p* < 0.001 in comparison: mock-infected vs. VSV-infected group (**A**); NC-treated vs. siRNA-treated groups after VSV infection (**B**); empty-vector-transfected vs. pFlag-pTRIM26-transfected after VSV infection (**C**).

Although we had shown that porTRIM26 plays a role in poly (I:C)-stimulated innate immune signaling, whether it plays a role in virus-triggered signaling remained to be determined. In previous studies, VSV has been used to study the effect of TRIM26 on IFN-β expression and antiviral response. Because PAM are resistant to VSV infection according to our preliminary experiments, we next investigated the effect of porTRIM26 (either positive or negative) on VSV infection in PIEC cells. Similar to poly (I:C) stimulation of PIEC cells, VSV infection also induced the expression of porTRIM26 in PIEC cells (Figure 4A). To identify the role of porTRIM26 in VSV infection we transfected PIEC cells with porTRIM26 siRNA, as described above. The knock-down of porTRIM26 expression significantly increased the expression of IFN-β during VSV infection compared with that in cells infected with the negative control. The viral titers were higher in the VSV infected-negative control cells than in VSV infected cells in which the expression of TRIM26 was knocked down (Figure 4B). To confirm these results, we transfected PIEC cells with the plasmid, as described above, to overexpress porTRIM26. ELISA data showed that the overexpression of porTRIM26 significantly reduced the expression of IFN-β relative to that in the VSV-infected empty-vector-transfected group. Notable, VSV infection was significantly greater in the porTRIM26-overexpressed cells than in the VSV-infected empty-vector-transfected cells. Taken together, these data suggest that porTRIM26 promotes viral infection by inhibiting IFN-β expression.

### 3.5. porTRIM26 Negatively Regulates Antiviral Response to PRRSV Infection

**Figure 5:**
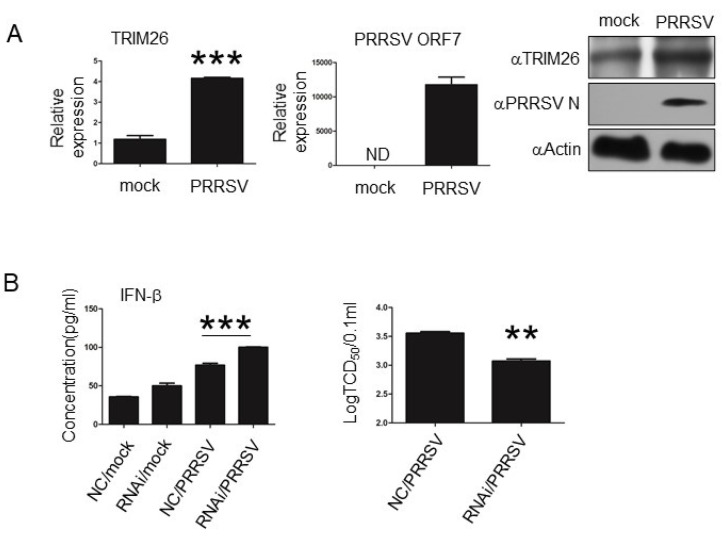
Effect of porTRIM26 on IFN-β expression and porcine reproductive and respiratory syndrome virus (PRRSV) infection in PAM. (**A**) PAM were infected with PRRSV at a MOI of 1 for 24 h. The mRNA levels of *TRIM26* and PRRSV *ORF7* were measured with RT-qPCR. The protein levels of TRIM26 and PRRSV N were measured with Western blotting. PRRSV infection induced expression of TRIM26. (**B**) PAM were transfected with the porTRIM26 siRNA or NC siRNA. After 72 h, cells were mock-infected or infected with PRRSV at an MOI of 1. Supernatants were collected at 24 hpi for either a TCID_50_ assay or ELISA. The knock-down of porTRIM26 expression increased the production of IFN-b and inhibited PRRSV infection. Data are mean ± SEM pooled from one independent experiment; *n* ≥ 3 for each of the analyzed parameters. ND: not detected. **, *p* < 0.01; ***, *p* < 0.001 in comparison: mock-infected vs. PRRSV-infected group (**A**); NC-siRNA-transfected vs. *porTRIM26*-siRNA-transfected cells after infection with PRRSV (**B**).

Our data showed that porTRIM26 inhibits IFN-β production and innate antiviral response. Therefore, we examined whether this also occurs during other viral infections. A previous study showed with an RNA-sequencing (RNA-seq) analysis that PRRSV infection could induce the expression of porTRIM26 [20], so we examined the biological functions of porTRIM26 using PRRSV, to which PAM are susceptible. Our data show that PRRSV infection induced the expression of TRIM26 in PAM (Figure 5A), consistent with the previous RNA-seq data. We also investigated the role of porTRIM26 in IFN-β production and the antiviral response in PRRSV-infected PAM. Our ELISA data showed that knocking down the expression of porTRIM26 increased the expression of IFN-β compared with that in the PRRSV-infected negative control (NC) cells (Figure 5B). We also noted that the viral titer was significantly lower in the PRRSV-infected porTRIM26-siRNA-transfected cells than in the PRRSV-infected negative control (NC) cells. These data suggest that PRRSV may inhibit IFN-β production and the antiviral response by inducing porTRIM26. These results confirm that porTRIM26 plays an inhibitory role in IFN-β expression and the antiviral response.

## 4. Discussion

Several studies have reported the gene sequence of *porTRIM26* based on genomic sequencing analyses [12,13]. However, the expression profiles of porTRIM26 in tissues and its biological functions have been unclear until now. In this study, we confirmed that porTRIM26 inhibits IFN-β production after poly (I:C) stimulation or viral infection. Using a rabbit anti-pTRIM26 polyclonal antibody generated with synthesized antigen peptide, we showed that poly (I:C) stimulation or viral infection (VSV or PRRSV) induces the expression of porTRIM26. By overexpressing or knocking down the expression of porTRIM26, we demonstrated that the induction of porTRIM26 modulates the IFN-β expression induced by poly (I:C) stimulation. Our data demonstrate that the induction of porTRIM26 negatively regulates IFN-β production and the antiviral response to VSV or PRRSV infection. Collectively, these data provide novel evidence that porTRIM26 modulates the innate antiviral response by inhibiting IFN-β production. 

Although more than 50 *TRIM* genes of the pig have been annotated, only TRIM21 has yet been functionally analyzed. Swine TRIM21 restricts foot-and-mouth disease virus (FMDV) infection with an intracellular neutralization mechanism [21]. Our data demonstrate that porTRIM26 negatively regulates IFN-β production and the antiviral response to PRRSV infection. These findings not only verify the role of porTRIM26 in IFN-β expression and the antiviral response but also extend our understanding of how PRRSV uses host proteins, such as porTRIM26, to interfere with the innate antiviral response. 

A previous study has shown that TRIM26 negatively regulates IFN-β production and the innate antiviral response by inhibiting activation of interferon regulatory factor 3 (IRF3) [15]. Because PRRSV infection interferes with the activation of IRF3, it cannot be excluded that PRRSV infection inhibits the activation of IRF3 by inducing porTRIM26. The exact mechanism by which PRRSV inhibits IFN-β production via induction of porTRIM26 needs to be clarified in future research. 

Although TRIM26 negatively regulates IFN-β production and the innate antiviral response, one study has reported that TRIM26 has the opposite function, promoting IFN-β production and the innate antiviral response [14]. It is possible that the different experimental systems used, including different methods and viruses, have generated different results. For example, TRIM21 is reported to degrade IRF3, thus limiting the type I IFN response after Japanese encephalitis virus (JEV) infection [22]. In contrast, another report suggested that TRIM21 acts as a positive regulator of the IRF3 pathway during Sendai virus infection [23]. Whether or not porTRIM26 positively affects IFN-β production and the innate antiviral response needs to be investigated in other swine viruses. However, our results suggest that porTRIM26 modulates IFN-β production and the innate antiviral response. 

In summary, this is the first study to identify the biological functions of porTRIM26. We have demonstrated that porTRIM26 plays an inhibitory role in IFN-β expression and the innate antiviral response. These findings extend our understanding of how some swine viruses, such as PRRSV, inhibit IFN-β production to evade the host’s innate immune response.

## Supplementary Materials

The following are available online at https://www.mdpi.com/2073-4425/11/10/1226/s1, Table S1: PCR primer used in the study.
