# Supplementary material for: Expression Profile of Porcine TRIM26 and Its Inhibitory Effect on Interferon-β Production and Antiviral Response
Source: Genes (Basel). 2020 Oct 19;11(10):1226. doi: 10.3390/genes11101226 (PMC7589756; doi:10.3390/genes11101226)
Supplement: Supplementary file 1 [file genes-11-01226-s001.pdf]

Table S1

PCR primer used in the study.

Primer	Sequence (5'-3')	Used for
pTRIM26-F	CGAAGCTTATGGCCACGTCAGCGCCAC (*Hin*dIII)	Gene amplification
pTRIM26-R	AAGGATCCGGGTCTCAGCAGGAGGCGTG (*Bam*HI)	Gene amplification
pTRIM26-F	TCTGGCAAAGTGGACATT	qPCR
pTRIM26-R	GGTGGAATCATACTGGAACA	qPCR
pIFN-β-F	ATCTTCGGCATTCTCAGA	qPCR
pIFN-β-R	CCATCAAGTTCCACAAGG	qPCR
VSV N-F	ACGGCGTACTTCCAGATGG	qPCR
VSV N-R	CTCGGTTCAAGATCCAGGT	qPCR
PRRSV ORF7-F	ATCCAGACTGCCTTCAAT	qPCR
PRRSV ORF7-R	AACTCCACAGTGTAACTTATC	qPCR
pGAPDH-F	TCTGGCAAAGTGGACATT	qPCR
pGAPDH-R	GGTGGAATCATACTGGAACA	qPCR
